# Efficacy and safety of Shenqi Fuzheng injection combined with platinum-based chemotherapy for stage III/IV non-small cell lung cancer: A protocol for systematic review and meta-analysis of randomized controlled trials

**DOI:** 10.1097/MD.0000000000017350

**Published:** 2019-09-27

**Authors:** Hongwei Chen, Xiaojun Yao, Zhengtang Liu, Ting Li, Cong Xu, Jue Wang, Xinbing Sui, Elaine Lai-Han Leung, Qibiao Wu

**Affiliations:** a State Key Laboratory of Quality Research in Chinese Medicine; b Faculty of Medicine; c Faculty of Chinese Medicine, Macau University of Science and Technology, Macau; d Department of Geriatrics, Xiyuan Hospital of China Academy of Chinese Medical Sciences, Beijing; e Department of Medical Oncology, Holistic Integrative Oncology Institutes and Holistic Integrative Cancer Center of Traditional Chinese and Western Medicine, the Affiliated Hospital of Hangzhou Normal University; f Department of Cancer Pharmacology, Holistic Integrative Pharmacy Institutes, College of Medicine, Hangzhou Normal University, Hangzhou, Zhejiang, P. R. China.

**Keywords:** efficacy, meta-analysis, non-small cell lung cancer, platinum-based chemotherapy, randomized controlled trial, safety, Shenqi Fuzheng injection, systematic review

## Abstract

**Background:**

Shenqi Fuzheng injection (SFI) is a commonly used anti-cancer Chinese patent medicine and has long been prescribed as adjunctive treatment to platinum-based chemotherapy (PBC) in patients with stage III/IV non-small cell lung cancer (NSCLC). However, the efficacy and safety of this combination therapy remain unclear.

**Methods:**

A systematic review and meta-analysis will be conducted following the Preferred Reported Items for Systematic Review and Meta-analysis (PRISMA) guidelines. Seven databases will be searched for relevant studies from their inception to the present date: PubMed, Web of Science, Cochrane Library, EMBASE, ClinicalTrials.gov, China National Knowledge Infrastructure (CNKI), and Wanfang Databases. All randomized clinical trials comparing SFI in combination with PBC versus PBC alone will be retrieved and assessed for inclusion. Two researchers will independently perform the selection of the studies, data extraction, and synthesis. The Cochrane Risk of Bias Tool will be used to evaluate the risk of bias of the RCTs. The primary endpoint is the disease control rate (DCR), the secondary outcomes are the objective response rate (ORR), survival rate, quality of life (QOL), cellular immune function, and toxicities. Review Manager 5.3 (Nordic Cochrane Centre, Cochrane Collaboration, 2014 Copenhagen, Denmark) will be used to analyze the outcomes.

**Results:**

This study will systematically evaluate the efficacy and safety of SFI combined with platinum-based chemotherapy in the treatment of stage III/IV NSCLC. The results will be published in a peer-reviewed journal.

**Conclusion:**

This systematic review will evaluate the effects of SFI as adjunctive treatment to platinum-based chemotherapy in the patients with stage III/IV non-small cell lung cancer, thus providing evidence to the clinical application of this combination therapy.

**PROSPERO registration number:**

CRD42019137196

## 1 Introduction

Lung cancer is the most common cancer and the leading cause of cancer-related deaths in the world and in China.^[1–4]^ More than 2.1 million people each year are diagnosed with lung cancer, 85% of them are non-small-cell lung cancers (NSCLC)^[5,6]^ and approximately two-thirds of lung cancers are diagnosed at stage III/IV.^[7]^ Although targeted therapy and immunotherapy have significantly improved the clinical outcomes of advanced lung cancer patients, many patients cannot benefit from these precision therapies because they lack an actionable biomarker or have no access to the precision therapies, for those patients, platinum-based chemotherapy (PBC) is still a commonly recommended treatment choice.^[8–10]^ Unfortunately, compared with the patients treated with precision therapies, those treated with PBC usually have lower objective tumor response, worse prognosis, poor quality of life (QOL), and increased risk of chemotherapy-induced toxic effects.^[11–15]^ Therefore, it is very important to find more optimal treatment regimens that can help to improve efficacy and alleviate the toxic effects of PBC treatment for advanced lung cancer patients.

In China, traditional Chinese medicine has been extensively used in the treatment of advanced NSCLC. Shenqi Fuzheng injection (SFI) is an important Chinese patent medicine (Drug Approval Number: Z19990065, China Food and Drug Administration) which is composed of Dangshen **(***Codonopsis pilosula*) and Huangqi (*Astragalus membranaceus*) and has been widely used as an adjunctive therapy to chemotherapy in the treatment of various cancers, including lung cancer, breast cancer, gastric cancer, and colorectal cancer, etc.^[16–22]^

The bioactive components of SFI include syringin, calycosin-7-O-β-D-glucopyranoside, lobetyolin, ononin, and astragaloside IV, etc.^[23,24]^ Increasing studies have shown that http://www.theplantlist.org/tpl1.1/record/kew-235193 SFI has antineoplastic properties, including inhibiting cancer growth, promoting apoptosis, increasing chemotherapy sensitivity, and improving immune functions, etc.^[25–29]^

Some clinical trials and a meta-analysis have already evaluated the effects of SFI combined with chemotherapy, indicating that SFI plus chemotherapy might improve the efficacy, immune function, and reduce adverse events in NSCLC.^[30–33]^ However, the efficacy and safety of SFI plus PBC for patients with stage III/IV NSCLC have never been systematically evaluated. The objective of this systematic review is to assess the effects of SFI plus PBC for stage III/IV NSCLC.

## 2 Method

### 2.1 Study registration

This study has been registered as PROSPERO CRD42019137196 (https://www.crd.york.ac.uk/prospero/display_record.php?RecordID=137196). Ethical approval is not required because all the research materials are published studies.

### 2.2 Criteria for considering studies for this review

#### 2.2.1 Types of studies

All relevant prospective clinical trials such as randomized clinical trials, controlled trials will be included in this study. Retrospective studies and non-RCTs will be excluded.

#### 2.2.2 Types of participants

Patients with a clear diagnosis of stage III/IV NSCLC, the age, sex, and ethnicity of the patients are not limited.

#### 2.2.3 Types of interventions

The intervention in the experimental groups is SFI combined with PBC. The intervention in the control groups is PBC alone.

#### 2.2.4 Types of outcome measures

Primary outcomes will be disease control rate (DCR), and secondary outcomes will be the objective response rate (ORR), QOL, cellular immune function, and toxicities.

### 2.3 Search methods for the identification of studies

#### 2.3.1 Search strategy

Two independent reviewers (HWC and XJY) will carry out a comprehensive search of the PubMed, Medline, Web of Science, Cochrane Library, EMBASE, China National Knowledge Infrastructure (CNKI), Wanfang Databases. The last search date will be Sept. 30, 2019. The strategy for searching in English databases and Chinese databases has been presented as an example in Table 1. Besides, we also searched and assessed the relevant systematic reviews and meta-analyses, aimed at finding the potential studies from their references.

**Table 1:**
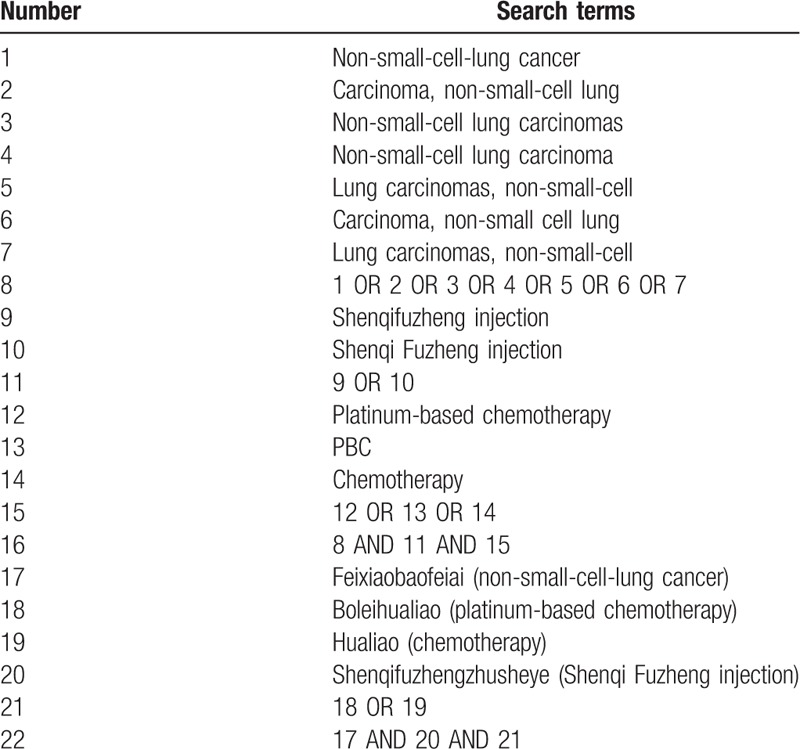
Search strategy applied in English databases (PubMed, Medline, EMBASE, etc) and search strategy applied in Chinese databases (China National Knowledge Infrastructure [CNKI], Wanfang Databases).

#### 2.3.2 Study selection

Two reviewers (HWC and XJY) will independently select relevant and inclusive articles by screening article titles and abstracts. Any disagreements will be resolved by consensus.

#### 2.3.3 Data collection and management

Two reviewers (HWC and ZTL) will independently evaluate the included RCTs and extract the data. We will use the intention-to-treat (ITT) analysis to analyze the results whenever available. We will extract all information from each report such as the authors, date of publication, countries, participants, interventions, outcomes, and results, study methods, etc. Any disagreement will be resolved by discussing with a third reviewer (QBW).

### 2.4 Assessment of risk of bias in included studies

Following the “Risk of Bias Assessment Tool” of the Cochrane Handbook for Randomized Controlled Trials,^[34]^ 2 researchers will independently assess the methodological treatment of the included literature. The risk of biases will be evaluated by some contents such as sequence generation, allocation concealment, blinding, incomplete outcome data, and selective outcome reporting, etc.

#### 2.4.1 Measurement of treatment effect

For dichotomous data, risk ratio (RR), odds ratio (OR), or hazard ratio (HR) with their 95% confidence intervals (95% CIs) will be showed. For continuous data, weighted mean difference (WMD) or standardized mean difference (SMD) with their 95% confidence intervals (95% CIs) will be presented.

#### 2.4.2 Assessment of heterogeneity

The *I*^2^ statistic will be used to determine the heterogeneity. If *I*^2^ < 50%, heterogeneity is regarded as minor. If *I*^2^ > 50%, heterogeneity is regard as substantial. When heterogeneity is observed, subgroup analysis will be performed to identify the possible causes.^[34,35]^

#### 2.4.3 Data synthesis

If heterogeneity is minor, a fixed-effects model will be used to estimate the summary RR (OR or RD), WMD (or SMD) and their 95% CIs, and meta-analysis will be carried out; if heterogeneity is substantial, a random-effects model will be used for data pooling, and meta-analysis will be conducted. If quantitative synthesis is not appropriate, a systematic narrative synthesis will be provided with the information presented to summarize and explain the characteristics and findings of the included studies.^[36]^ The strength of the body of evidence will be judged using the Grading of Recommendations Assessment, Development and Evaluation working group methodology (GRADE).^[37]^

#### 2.4.4 Risk of bias across trials

Funnel plots will be used to detect reporting biases. When the number of the included trials is >10, Begg tests, Egger test, and funnel plots will be used to examine the potential bias in the RCTs included in the meta-analysis.^[38,39]^

#### 2.4.5 Additional analyses

When heterogeneity is significant, subgroup analysis will be used to determine the possible causes such as sample size, age, sex, drug dose, dosage form, and course of treatment, etc. Sensitivity analysis, subgroup analysis will be used to determine the robustness of results. A meta-regression analysis will be performed to test the potential heterogeneity.

## 3 Discussion

Currently, there is no published systematic review and meta-analysis evaluating the efficacy and safety of SFI plus PBC for stage III/IV NSCLC, this review will provide valuable evidence to this combination therapy for the patients with advanced NSCLC.

## Author contributions

QBW, WZ, and LHL conceived and designed the study, revised the manuscript, HWC, XJY, and ZTL developed the criteria and performed literature research, and wrote the protocol, TL, CX, and JW advised on protocol design and revised the manuscript. All authors read and approved the final manuscript. QBW is the guarantor of the review.

Hongwei Chen orcid: 0000-0001-9010-9728.

Xiaojun Yao orcid: 0000-0002-6972-2971.

Zhengtang Liu orcid: 0000-0002-1155-8195.

Ting Li orcid: 0000-0003-4990-5997.

Cong Xu orcid: 0000-0002-9110-4600.

Jue Wang orcid: 0000-0002-6151-1117.

Xinbing Sui orcid: 0000-0001-7330-0467.

Elaine Lai-Han Leung orcid: 0000-0002-3705-8084.

Qibiao Wu orcid: 0000-0002-1670-1050.
